# Phosphatases in tumor cell immune escape: a perspective based on the camouflage, coercion, cytoprotection

**DOI:** 10.3389/fcell.2026.1645868

**Published:** 2026-02-19

**Authors:** Chencheng Zhang, Xinyue Qiu, Weidong Shi, Shudong Zhu

**Affiliations:** 1 Cancer Research Center, Nantong Tumor Hospital, Nantong, China; 2 Cancer Research Institute, The Affiliated Tumor Hospital of Nantong University, Nantong, China; 3 Cancer Research Center, Nantong, China; 4 Department of Oncology, Nantong Tumor Hospital, Nantong, China; 5 Department of Thoracic Surgery, The Second People Hospital of Nantong, Nantong, China

**Keywords:** “three Cs” framework, antigen presentation, immune checkpoint, metabolic reprogramming, phosphatases, targeted therapy, tumor immune escape

## Abstract

Tumor immune evasion represents a core challenge restricting the efficacy of cancer treatment, and a deep understanding of its underlying mechanisms is crucial for developing novel immunotherapeutic approaches. This article focuses on the multidimensional regulatory roles of protein phosphatases in this critical biological process, innovatively adopting the “three Cs” framework—*camouflage, coercion, and cytoprotection*—for systematic elaboration, thereby revealing phosphatases as core molecular switches within dynamic regulatory networks. Our review systematically demonstrates that protein phosphatases serve as indispensable “dynamic molecular switches” within the “three Cs” framework of tumor immune evasion. Their regulatory networks span the entire continuum of tumor cells evading recognition, inhibiting immune cell function, and resisting terminal immune attacks. This insight underscores the substantial potential of targeting phosphatase regulatory networks, which may overcome the drug resistance bottleneck encountered in current immunotherapies. By designing novel drug strategies to precisely intervene in key phosphatase nodes—thereby achieving “one target, multiple effects” synergistic regulation—this framework provides a robust theoretical foundation and promising new avenues for developing more efficient, broad-spectrum next-generation tumor immunotherapies.

## 1 Introduction

Immune evasion in cancer represents one of the core characteristics of tumorigenesis, progression, and therapeutic resistance. With the advancement of immunotherapy, a profound understanding of immune evasion mechanisms has gained paramount importance. The traditional “three Es” model (Elimination, Equilibrium, and Escape), proposed by Dunn et al., in 2002, provides a crucial conceptual framework for understanding tumor-immune dynamics (Dunn et al., 2002). The Three Es model linearly describes how the immune system eliminates malignant cell precursors, maintains dynamic equilibrium with microscopic tumors, and how tumor cells subsequently acquire genetic or epigenetic alterations to achieve immune evasion. However, this model primarily describes the macroscopic process of immunoediting and fails to thoroughly reveal the specific strategies employed by tumor cells to execute immune escape at the molecular level. Consequently, it is difficult to systematically elucidate how tumor cells actively regulate key processes such as immune recognition, immune attack, and their own survival.

To make up for the above shortcomings, Claudia Galassi et al. innovatively proposed the “three Cs” framework (Camouflage, Coercion, Cytoprotection) in 2024 (Galassi et al., 2024). This framework provides a comprehensive classification and in-depth analysis of tumor cell behavior: Camouflage elucidates how tumor cells hide from immune detection; Coercion describes how tumor cells actively suppress immune cell function; Cytoprotection focuses on how tumor cells resist immune-mediated killing to enhance survival. The “three Cs” framework addresses tumor immune evasion across three dimensions: spatial (evasion of immune recognition), functional (suppression of immune cells), and survival (anti-cytotoxicity) - thereby more accurately reflecting the dynamic complexity of tumor-immune interactions. Therefore, placing molecular mechanisms within the “three Cs” framework for systematic integration has significant theoretical innovation potential.

Phosphatases, as key regulatory molecules in intracellular signal transduction, are widely involved in multiple processes including immune recognition, immune cell activity, and tumor cell survival by precisely regulating protein phosphorylation status (Song et al., 2022; Khokhlatchev et al., 1998; Lin et al., 2024). Classified primarily by catalytic domains and substrate specificity, phosphatases comprise families such as protein tyrosine phosphatases (PTPs), serine/threonine phosphatases (PPPs, e.g., PP1, PP2A), and dual-specificity phosphatases (DUSPs) (Tonks, 2006; Caunt and Keyse, 2013; Janssens and Goris, 2001; Cohen, 1989). PTP affects tumor cell immune escape by regulating processes such as antigen presentation and tumor microenvironment (Linghu et al., 2025; Qu et al., 2023). PPPs maintain genomic stability through dephosphorylation of cyclin-dependent kinases (CDKs) or apoptotic regulators (e.g., BAD) (Chiang et al., 2001; Ambjørn et al., 2021; Wei et al., 2013). DUSPs negatively regulate MAPK pathways (e.g., ERK, p38) via dual-specific dephosphorylation of tyrosine and serine/threonine residues, influencing tumor cell plasticity (Png et al., 2024; Ferguson et al., 2019; Ramkissoon et al., 2019). Although multiple studies have suggested that phosphatases play a crucial role in immune regulation, current research predominantly focuses on individual molecules or pathways, lacking a holistic view that systematically elucidates how phosphatases coordinate different immune evasion modules within a unified framework.

Based on this, this study adopts the “three Cs” model as a systemic blueprint to comprehensively map, for the first time, the functional network of phosphatases throughout the entire process of tumor immune evasion: exploring how they assist tumor cells in evading immune recognition during “camouflage”; how they participate in suppressing immune cell function under “coercion”; and how they enhance tumor cell resistance to immune-mediated attacks during “cellular protection”. Through this integrated perspective, this study not only addresses the mechanistic gaps in the traditional “three Es” model but also overcomes the “fragmented and isolated” limitations of previous phosphatase research. It provides a novel theoretical foundation for developing “single-target, multi-effect” immunotherapy strategies targeting phosphatases.

## 2 Phosphatases in camouflage

### 2.1 Antigen processing and presentation defects

Antigen Processing and Presentation (APP) is a fundamental process for the immune system to recognize and eliminate abnormal cells. This process is primarily mediated by Major Histocompatibility Complex (MHC) molecules: MHC class I molecules, ubiquitously expressed on nucleated cell surfaces, present endogenous or intracellular peptides for recognition by CD8^+^ (cytotoxic) T cells; MHC class II molecules, predominantly expressed on Antigen Presenting Cells (APCs), present exogenous antigens to CD4^+^ T cells (Pishesha et al., 2022; Roche and Furuta, 2015). APP process defects involving multi-faceted pathways: genetic variations, epigenetic modifications, loss of neoantigen expression, dysregulation of post-translational modifications, and compromised protein stability (Burr et al., 2019). Notably, Defects in the APP represent a critical strategy by which tumors achieve immune “camouflage” and evade immune surveillance, with phosphatases precisely regulating this process through various molecular mechanisms.

Firstly, phosphatases are involved in regulating the gene expression and protein stability of MHC class I (MHC-I). Mutations in MHC molecules and their chaperone protein β2-microglobulin (B2M) disrupt cell-surface MHC-I expression, thereby abolishing the ability of tumor cells to present antigens to CD8^+^ T cells. On one hand, histone methyltransferase EZH2 can inhibit MHC-I gene transcription by catalyzing the deposition of H3K27me3, leading to epigenetic silencing (Lehmann et al., 2021; Romero et al., 2024)^.^ During this process, the serine/threonine phosphatase PP1 enhances the methyltransferase activity of EZH2 through dephosphorylation at the S21 site, thereby indirectly suppressing MHC-I expression (Zhang et al., 2022). On the other hand, the transcription factor MITF inhibits MHC-I translation by directly binding to and repressing the promoter of eukaryotic initiation factor 3B (Santasusagna et al., 2023). The tyrosine phosphatase SHP2 promotes MITF ubiquitination and degradation through dephosphorylation at its S73 site, thereby indirectly maintaining MHC-I translation levels (Bhattacharyya et al., 2016; Liu X. et al., 2020). The SUSD6-TMEM127-WWP2 complex mediates MHC-I ubiquitination and lysosomal degradation, a process also indirectly regulated by the phosphatase network (Chen X. et al., 2023; Wang et al., 2022).

Secondly, phosphatases participate in interfering with the interferon-γ (IFN-γ) signaling pathway. IFN-γ is a key cytokine that induces the expression of MHC-I and antigen processing-related molecules. The increased activity of phosphatase SHP1 inhibits the expression of STAT1-dependent immune escape-related molecules by reducing the IFN-γ-mediated tyrosine phosphorylation of JAK1/2 and STAT1 (Ye et al., 2023). The tyrosine phosphatase PTPN2 directly inhibits IFN-γ signal transduction by dephosphorylating JAK1 and STAT1, thereby blocking MHC-I upregulation (An et al., 2025). Notably, PTPN2 deficiency enhances the therapeutic efficacy of PD-1 blockade in melanoma (Luo et al., 2018). Additionally, PP2A indirectly suppresses MHC-I expression by enhancing TCF4 transcriptional activity through indirect degradation of β-catenin (Luo et al., 2024; Hoehn et al., 2015).

Finally, phosphatases are involved in coordinating compensatory immunosuppressive signals. When MHC-I expression is downregulated in tumor cells, other inhibitory ligands are simultaneously upregulated to compensate and suppress the activity of innate immune cells (Zheng et al., 2023). The antitumor cytotoxicity of natural killer (NK) cells is tightly regulated by MHC-I-specific inhibitory receptors on their surface (Wu et al., 2021). When the inhibitory receptor NKG2A on NK cells binds to the non-classical MHC-I molecule HLA-E on tumor cells, the immunoreceptor tyrosine-based inhibitory motif (ITIM) in the intracellular domain of NKG2A is phosphorylated by Src family kinases. This phosphorylation subsequently recruits the tyrosine phosphatases SHP-1/2. SHP-1 transmits inhibitory signals by dephosphorylating key signaling molecules such as VAV1, thereby counteracting the activation state of NK cells and suppressing their cytotoxic function (Borst et al., 2020; Motoda et al., 2000). Upregulation of tyrosine phosphatase SHP-1 (PTPN6) expression and phosphorylation specifically inhibits STAT3 and ERK phosphorylation, which partially restores NK cell function (Yu et al., 2025). Furthermore, tumor cells highly express CD47, which transmits a “do not eat me” signal by binding to SIRPα on macrophages (Catalan et al., 2020). The phosphatase SHP2 is recruited to the ITIM domain of SIRPα and dephosphorylates it, thereby inducing dephosphorylation of myosin IIA and, therefore, inhibition of the cytoskeleton rearrangement, ultimately leading to impaired phagocytic function of macrophages (Catalan et al., 2020).

### 2.2 Inhibition of chemotaxis, migration, and function of antigen-presenting cells (APCs)

Through the aforementioned processes, tumor cells successfully interfere with the expression and function of MHC-I molecules, rendering cytotoxic T cells unable to effectively recognize them, thereby achieving “invisibility.” However, a small amount of antigen leakage still occurs during this process. Thus, tumor cells disrupt the recruitment and function of APCs, preventing them from presenting antigens to T cells and thereby interrupting the initiation of adaptive immunity. This process involves the disintegration of the chemokine network, the construction of an immunosuppressive chemical environment, interference with APC phagocytic recognition, and the establishment of physical and cellular barriers (Kim and Cho, 2022; Luri-Rey et al., 2025; Hsieh et al., 2022). Phosphatases play a central regulatory role in this process, collectively constructing a defensive line that inhibits immune cell infiltration through multiple mechanisms.

Chemokines primarily function by binding to G protein-coupled receptors (GPCRs) on the cell surface, activating downstream signaling pathways to guide the directional migration of immune cells. Tumor cells indirectly regulate the production of key chemokines (such as CXCL10 and CCL4) through phosphatases, preventing APCs from receiving correct “navigation” signals. Specifically, the phosphatase SHP-1 dephosphorylates STAT1 (Tyr701), blocking the IFN-γ signaling pathway and thereby inhibiting the production of its downstream key chemokine CXCL10 (Xu et al., 2018; Metwally et al., 2025; Feng et al., 2002; Shi et al., 2021). PP2A inhibits NF-κB through the AKT pathway, downregulating CXCL10 expression (Liu et al., 2005; Sanz-Castillo et al., 2023). Meanwhile, PP1 cooperates with histone deacetylase HDAC8 to silence the CCL4 promoter mediated by the transcription factor CREB (Gao et al., 2009; Yan et al., 2024). These actions collectively result in insufficient secretion of chemokines essential for APC recruitment, jointly impairing the recruitment of cDC1 and T cells (Yan et al., 2024; Bystry et al., 2001).

In addition, tumor cells actively suppress the migratory function of APCs by generating immunosuppressive molecules. The triphosphate diphosphohydrolase (NTPDase) CD39/CD73 hydrolyze extracellular ATP into adenosine (Rodríguez-Martínez et al., 2025). Adenosine not only acts as an immunosuppressive molecule itself but also binds to the A2A receptor on APCs, activating serine/threonine protein phosphatase activity (Revan et al., 1996). PP2A directly inhibits APC motility by dephosphorylating the S239 and S157 sites of the migratory-related protein VASP, forming a migratory inhibition loop (Kumm et al., 2020; Estin et al., 2017).

Next, tumor cells evade phagocytosis by APCs through bidirectional regulation of phagocytic signals. Endoplasmic reticulum (ER) stress is crucial for inducing the membrane translocation of calreticulin (CALR), the “eat-me” signal (Banuelos et al., 2025). Phosphatases PP1/PP2A block ER stress by dephosphorylating eIF2α at S51, thereby inhibiting CALR membrane translocation (Santos et al., 2016; Placido et al., 2017; Beltrán-Visiedo et al., 2024; Liu P. et al., 2020). Meanwhile, tumor cells overexpress CD47, which binds to SIRPα on APCs and recruits phosphatases SHP-1/SHP-2 (Oldenborg et al., 2001; Ma et al., 2024). These phosphatases block phagocytic synapse formation by dephosphorylating phagocytic signal proteins Vav/Syk, the “don’t-eat-me” signal (Sohn et al., 2011; Miller et al., 2025). Phosphatase PP2A may also promote tumor immune escape by inhibiting the cGAS-STING-IFN pathway (Mondal et al., 2023). These two processes synergistically achieve dual evasion: defective “eat-me” signals and overactive “don’t-eat-me” signals.

Finally, tumor cells isolate APCs by constructing physical and cellular barriers (Papadas et al., 2023). The loss of phosphatase PPM1A leads to sustained phosphorylation of Smad2/3, promoting the activation of cancer-associated fibroblasts (CAFs), and the TGF-β1 secreted by CAFs drives collagen deposition and extracellular matrix (ECM) remodeling (e.g., DDR1-mediated parallel collagen arrangement) (Hong et al., 2021; Liang et al., 2023; Kim et al., 2019). DUSP1 inhibits the secretion of matrix metalloproteinases (MMPs) by dephosphorylating p38 MAPK, thereby reducing ECM degradation and jointly reinforcing the physical barrier (Zhang et al., 2018; He T. et al., 2025). PP2A dephosphorylates LATS1/2 kinases, activating the YAP transcriptional program to promote dense collagen production and reinforce the physical barrier (Hein et al., 2019; Liao et al., 2025; Ribeiro et al., 2010). Phosphatases SHP-1 and CD45 maintain the survival and function of tumor-associated macrophages (TAMs) and myeloid-derived suppressor cells (MDSCs), respectively, by regulating CSF1R and STAT3 signaling, thereby forming an immunosuppressive cellular barrier (Wei et al., 2024; He J. et al., 2025; Ramesh et al., 2019; Hong et al., 2023).

### 2.3 Summary

#### 2.3.1 Core mechanism

Phosphatases orchestrate a multi-layered, synergistic regulatory network spanning molecular (antigen), signaling (chemokine), and tissue (physical/cellular barrier) levels, thereby dominating tumor immune evasion. The essence lies in achieving simultaneous “self-concealment” and “environmental blockade.”

#### 2.3.2 Existing controversies and challenges

The greatest challenge stems from the cellular specificity and network complexity of phosphatase functions. The same phosphatase (e.g., PP2A, SHP-1) may exert opposing roles in tumor cells versus immune cells, while cross-talk among phosphatase pathways exacerbates off-target effects in targeted therapies. Furthermore, the hierarchical regulation and dynamic evolution of distinct evasion mechanisms remain poorly defined.

#### 2.3.3 Therapeutic opportunities

Therapeutic strategies targeting this network should focus on “dismantling immune evasion.” Potential approaches include:

Combinatorial targeting, such as pairing PTPN2 inhibition with PD-1 blockade to restore MHC-I expression and T-cell functionality; Multi-node intervention, such as simultaneously targeting CD39/CD73 (to relieve chemical suppression) and SHP2 (to alleviate phagocytic and signaling inhibition), which may generate synergistic effects to fully reverse the tumor’s immunosuppressive microenvironment.

## 3 Phosphatases in coercion

When the “camouflage” of tumor cells is partially uncovered, they will adopt more proactive “coercion” strategies, directly suppressing or disrupting the functions of immune effector cells while fostering the expansion of immunosuppressive cells, thereby functionally dismantling the anti-tumor immune response (Galassi et al., 2024; Schreiber et al., 2011). Phosphatases act as key effectors in this process by modulating immune checkpoints, innate immune signaling pathways, cytokine networks, and metabolic pathways (Low and Zhang, 2016; Shi et al., 2016; Yi and Lindner, 2008).

### 3.1 Manipulating immune checkpoints and co-stimulatory signals

Immune checkpoint molecules, such as programmed cell death protein 1 (PD-L1), serve as the core weapons employed by tumor cells to exert immunosuppressive pressure. The expression level of these molecules on the cell membrane surface directly determines the intensity of immunosuppression. In regulating immune-modulating ligand expression, phosphatases directly dampen tumor cell immunogenicity by modulating immune checkpoint molecules and antigen presentation-related proteins (Mondal et al., 2023; Triolo et al., 2022). PTPN2 can reduce the expression abundance of PD-L1, in triple-negative breast cancer, PTPN2 deletion increases PD-L1 stability, enhancing T cell inhibition (Tang et al., 2023; Goh et al., 2022). This process is driven by IL-6/JAK1 signaling, where JAK1 phosphorylates PD-L1 and PTPN2 counteracts this via dephosphorylation (Chan et al., 2019; Kleppe et al., 2011).

Simultaneously, phosphatases influence the antigen presentation mechanism of tumor cells by participating in the regulation of MHC-I gene expression and protein stability. This function has been elaborated in detail in the preceding section on “camouflage” and will not be reiterated here. Furthermore, phosphatases evade anti-tumor immunity by suppressing co-stimulation signaling pathways within the immune system, thereby inhibiting the activity of immune cells (Baumgartner et al., 2023). The immune effector cells involved include, but are not limited to, dendritic cells (DCs), NK cells, TH1-polarized CD4^+^ T cells, and CD8^+^ cytotoxic T lymphocytes (CTLs) (Armitage et al., 2021; Spalinger et al., 2016). Additionally, phosphatases promote the activity of immunosuppressive cells, such as regulatory T (TReg) cells, specific TAMs subsets, and MDSCs (Schreiber et al., 2011; Moon et al., 2014). For instance, SHP1/SHP2 dephosphorylates the CD80 molecules on the surface of DCs, consequently reducing the intensity of CD80/CD86:CD28 co-stimulation signaling (Xie et al., 2023; Sharma A. et al., 2017). In melanoma models, DCs overexpressing SHP1 exhibit significantly impaired T-cell activation capacity (Matous et al., 2025).

### 3.2 Disrupting the sensing of innate immunity and interferon signaling

The interferon signaling pathway, particularly the production and response of Type I interferons, serves as a critical bridge linking innate immunity with adaptive immunity and is essential for initiating an effective anti-tumor immune response (Zannikou et al., 2024; Diamond et al., 2011). Phosphatases act as a key tool to disrupt this bridge, as tumor cells exploit phosphatases to sabotage the immune system’s “alarm” mechanism, thereby preventing the initiation of an effective immune response.

In the cGAS-STING-TBK1/IRF3 pathway, the phosphatase SHP2 directly binds to the kinase domain of TBK1 through its C-terminal domain (residues 273-538, encompassing the PTP domain), inhibiting its kinase activity and consequently blocking the activation of downstream IRF3 (Decout et al., 2021; Qiao et al., 2021; Zhao et al., 2019; Gao et al., 2022). Concurrently, studies have demonstrated that SHP2 can function as a scaffold to bridge DAP12 and TRIM27, mediating the K48 ubiquitination of TBK1 by TRIM27 (Zheng et al., 2015). This subsequently leads to proteasomal degradation of TBK1, inhibits the phosphorylation of IRF3, and ultimately suppresses the production of type I interferons. The phosphatase PP2A dephosphorylates Carma1, rendering it incapable of recruiting key mediators for T-cell activation, which consequently restricts the production of IL-2 and IFN-γ (Eitelhuber et al., 2011). The deficiency of PP2A/STRN4 in macrophages reduces YAP/TAZ expression and renders tumor-conditioned macrophages sensitive to STING activation (Ho et al., 2023). PP2A can also dephosphorylate and inactivate the S172 site of TBK1, weakening nucleic acid sensing signal transduction and the anti-tumor immunity it initiates (Meng et al., 2021). Besides, SHP2 and PP2A can also directly dephosphorylate JAK kinases or STAT transcription factors downstream of interferon receptors, thereby interrupting the signaling of key cytokines such as IFN-γ (Shanker et al., 2013; Courdy et al., 2023; Liu et al., 2021). When IFN-γ secreted by T cells fails to effectively activate the JAK-STAT pathway in target cells, its induction of MHC molecule upregulation and anti-proliferative effects are stifled at their inception (Zhou, 2009). Clinical studies show PP2A overexpression significantly attenuates the anti-tumor effects of cGAMP (a STING agonist) (Pepin and Gantier, 2018). Additionally, SHP2 (PTPN11) interferes with ISGF3 complex (STAT1-STAT2-IRF9) assembly by dephosphorylating STAT1 at Y701, impairing IFN signaling, thereby blocking the transmission of type I and type II interferon signals (Luo et al., 2018; Roca-Rivada et al., 2025; Nowicka et al., 2023). In KRAS-mutant lung cancer, SHP2 inhibitors restore tumor cell IFN responsiveness, confirming its critical negative regulatory role in the IFN pathway (Mainardi et al., 2018; Christian et al., 2012). The blockade of interferon signaling leads to impaired expression of downstream stimulate genes, rendering tumor cells and their microenvironment “unresponsive” to the activating signals of the immune system.

### 3.3 Reprogramming the cytokine network

The balance of cytokines within the tumor microenvironment determines the direction of the immune response. Phosphatases, by remodeling this network, can shift a pro-inflammatory environment to an anti-inflammatory one (Yoon and Lee, 2022).

In the TGF-β pathway, phosphatase PPM1A (PP2Cα) promotes Smad2/3 nuclear translocation by dephosphorylating their C-terminal SXS motif, driving transcription of TGF-β target genes like chemokine CCL2 (Wrighton et al., 2006; Lin et al., 2006). This directly drives the differentiation and function of regulatory T cells and promotes epithelial-mesenchymal transition, jointly creating a suppressive microenvironment (Sheng et al., 2025; Lien et al., 2025; Zhao et al., 2014). Within T cells, when inhibitory receptors (such as PD-1) are engaged, their ITSM (Immunoreceptor Tyrosine-Based Switch Motif) motifs recruit SHP2 (or, in some cases, SHP1) (Kondo et al., 2025; LI et al., 2025). Subsequently, SHP2 becomes activated and relocates to the vicinity of the T cell receptor (TCR) signaling complex, where it dephosphorylates phosphorylated tyrosine residues on key signaling molecules such as ZAP70 and the CD3ζ chain (Sheppard et al., 2004; Lee et al., 2008). This directly truncates the activation signals in T cells, leading to their functional inactivation, cessation of proliferation, and even progression toward exhaustion (Hui et al., 2017). This represents the most direct molecular manifestation of tumor cells “coercing” T cells.

In natural immune cells, SHP1 (PTPN6) inhibits macrophage secretion of proinflammatory factors (e.g., TNF-α, IL-1β) by dephosphorylating p38 MAPK and blocking downstream MK2 kinase activation (Yang et al., 2019; He et al., 2020). Studies show SHP1 synergizes with IL-10 signaling to amplify the anti-inflammatory response (Minter et al., 2000). The dual-specificity phosphatase (DUSP) family is responsible for dephosphorylating Thr and Tyr residues within the MAPK pathway. For instance, DUSP 1 has been shown to be an important negative regulator of inflammatory response, affecting the production of pro-inflammatory and anti-inflammatory cytokines by modulating the p38 and Jun N-terminal protein kinase (JNK) MAP kinase pathways (Cornell et al., 2010). However, DUSP4 targets the mechanism of ERK inactivation, thereby reducing DUSP1 expression and eliminating its negative inhibition on cytokine production (Cornell et al., 2010). The role of DUSPs in tumor immunity is controversial (Keyse, 2008). DUSP4 can play a role in maintaining high ERK1/2 activity by negatively regulating DUSP6, thereby contributing to the survival and growth of melanoma cells (Kamada et al., 2022). The MAPK pathway not only affects the malignancy of tumor cells, but also affects the production and functional execution of immune cytokines (such as TNF-α and IL-2), thereby affecting the overall strength of the immune response (Kar et al., 2010).

### 3.4 Dominating metabolic reprogramming and nutrient deprivation

Tumor cells enhance their survival by altering metabolic landscapes, creating a microenvironment that directly suppresses effector immune cells while nourishing suppressive cells. Phosphatases serve as a crucial hub through which tumor cells orchestrate this metabolic reprogramming and impose “metabolic stress” on immune cells.

In tumor cells, SHP2 has been identified as a key node in multiple oncogenic signaling pathways (e.g., RAS-MAPK) (Liotti et al., 2021). Its activity drives the Warburg effect (aerobic glycolysis) in tumor cells, leading to substantial glucose consumption in the microenvironment and the production of copious amounts of lactate (Dong et al., 2023). For effector T cells, which primarily rely on aerobic glycolysis for energy, this results in severe glucose deprivation and impaired function (Longo et al., 2025). PTEN is typically regarded as a tumor suppressor gene, and its deletion or inactivation occurs frequently in various tumor types (Wang Q. et al., 2025). As a lipid phosphatase, PTEN’s core function is to catalyze the dephosphorylation of PIP3 to PIP2, thereby negatively regulating the potent pro-growth and metabolic signaling pathway, PI3K-AKT-mTOR (Liu et al., 2022). When PTEN is deleted, the PI3K signaling pathway becomes hyperactivated, significantly promoting glucose uptake and glycolysis in tumor cells and intensifying competition with T cells for glucose (Wang J. et al., 2025). Concurrently, uncontrolled PI3K signaling also drives predatory utilization of other nutrients, such as amino acids, collectively creating a metabolic environment unfavorable for T cell survival and function (Ramapriyan et al., 2019). ILKAP (a PP2C family phosphatase) stabilizes HIF-1α by dephosphorylating it, preventing VHL complex-mediated recognition and degradation (Liu et al., 2018). This upregulates genes like LDHA and GLUT1, promoting lactate production (Jin et al., 2022). A high-lactate environment can directly suppress the cytotoxicity and proliferation of T cells and NK cells (Ippolito et al., 2019). CD73 (NT5E), which possesses phosphatase activity, is a key enzyme involved in the generation of extracellular adenosine (Jackson et al., 2014). It catalyzes AMP hydrolysis to adenosine (Jackson et al., 2014; Bin et al., 2018). Adenosine activates T cell A2A receptors (ADORA2A), triggering PKA-CREB signaling to inhibit TCR signaling and cytokine production, and induce T cell dysfunction (Zhang et al., 2023). Targeting CD73 with inhibitors restores T cell function (Furriel et al., 2025; Liu et al., 2025). Additionally, the phosphatase SHP2 also enhances the enzymatic activity of IDO1 by dephosphorylating its Y115 and Y253 residues (Albini et al., 2017; Hoffka et al., 2025). This accelerates the conversion of tryptophan to kynurenine, leading, on one hand, to the depletion of tryptophan, which is essential for T cell activation, and, on the other hand, to the accumulation of kynurenine that can activate the aryl hydrocarbon receptor, directly driving the differentiation of regulatory T cells (Torres-Martínez et al., 2025). This dual mechanism exerts a suppressive effect on T cell immunity.

### 3.5 Summary

#### 3.5.1 Core mechanism

Phosphatases systematically impose immune “stress” by constructing a three-dimensional regulatory network that spans from membrane receptors to nuclear transcription and from intercellular communication to the metabolic environment. Their core characteristic lies in simultaneously suppressing the function of effector cells and fostering the population of suppressive cells, thereby establishing a multi-layered immunosuppressive system.

#### 3.5.2 Existing controversies and challenges

The primary challenge lies in the spatiotemporal specificity and cell-type specificity of phosphatase functions. For instance, SHP2 exhibits opposing regulatory effects in tumor cells and immune cells, potentially giving rise to complex network effects. Moreover, the synergistic interactions and dynamic evolutionary patterns among different stress mechanisms still require in-depth exploration.

#### 3.5.3 Therapeutic opportunities

Combination therapy strategies targeting the “stress” network demonstrate immense potential. Inhibition of PTPN2 can simultaneously enhance antigen presentation and reduce PD-L1 stability; the combination of SHP2 inhibitors with CD73 monoclonal antibodies can reverse multiple metabolic suppressions. Integrating these targeted drugs with existing immune checkpoint inhibitors holds promise for achieving synergistic anti-tumor effects. Currently, 17 related clinical trials are underway, offering a new direction to overcome current resistance to immunotherapy.

## 4 Phosphatases in cytoprotection

Cytoprotection constitutes the final defense line for cancers against immune attacks. This occurs when cancer cells, having been recognized and targeted by immune effector cells (such as CTLs or NK cells), resist immune-mediated elimination through mechanisms including cancer cell survival, immune exhaustion, and clonal evolution (Sharma P. et al., 2017; Kalbasi and Ribas, 2020; Mclane et al., 2019; Rosenthal et al., 2019). The phosphatase network plays a central role in this process by precisely regulating the dephosphorylation of key signaling molecules.

### 4.1 Reinforce physical barriers

Tumor cells interfere with the function of immunological synapses and membrane integrity by altering cytoskeletal structures and membrane repair capabilities, thereby physically obstructing the delivery of cytotoxic substances.

Phosphatase PP2A promotes actin depolymerization by dephosphorylating microtubule-actin crosslinking factor 1 (MACF1), reducing its stability (Wang et al., 2019). This enhances cell membrane flexibility, altering mechanical properties and decreasing membrane tension (Sousa and SOusa, 2021). This process destabilizes the immunological synapse structure formed by CTL/NK cells, effectively preventing the formation of functional pores by perforin on the target cell membrane and significantly blocking the influx of granzyme B (Wang et al., 2019; Lyubchenko et al., 2003). This physical barrier mechanism functionally antagonizes the phosphorylation regulation of FLNA by ROCK kinase, jointly and precisely regulating the intensity of the cytoskeletal response to immune attacks (Peverelli et al., 2018; Hong et al., 2024; Pan et al., 2021). Concurrently, the ER-localized tyrosine phosphatase PTP1B can promote the rapid recruitment and assembly of CHMP4B, a core component of the ESCRT-III membrane repair mechanism, at membrane damage sites caused by perforin by activating it (Elias et al., 2023; Kamenetsky et al., 2025). This complex actively internalizes and seals membrane pores through a budding process, effectively preventing the continuous penetration of granzymes (Gros et al., 2022). This mechanism is particularly prominent in melanoma and colorectal cancer, where membrane damage occurs frequently.

### 4.2 Interception of death signals

Tumor cells employ phosphatases to intervene at multiple nodes within death signaling pathways, including multi-tiered inhibition from receptors to core apoptotic pathways, thereby systematically reducing their sensitivity to immune-mediated apoptosis. On one hand, phosphatases participate in the suppression of death receptor pathways. The phosphatase SHP-1 disrupts the conformation of the death domain by directly dephosphorylating specific tyrosine residues in the intracellular domain of the FAS receptor, thereby blocking the recruitment of the FADD adapter protein (Koncz et al., 2008). This mechanism effectively inhibits the formation of the CASPASE-8 activation complex, strongly suppressing the transmission of extrinsic apoptotic signals even in tumor cells with high FAS receptor expression (Futosi et al., 2013; Daigle et al., 2002). In some solid tumors with intact FAS signaling pathways, this mechanism functionally complements the epigenetic silencing of the FAS gene, providing dual protection for tumor survival.

On the other hand, phosphatases can interfere with cytokine-induced apoptosis. Phosphatases PTPN2 and PTPN1B effectively disrupt the JAK-STAT signaling pathway induced by cytokines such as IFN-γ by dephosphorylating JAK1 and JAK2 (Bourdeau et al., 2005; Pike and Tremblay, 2016; Perez-Quintero et al., 2025). This not only diminishes the transcriptional activity of STAT1, leading to the downregulation of pro-apoptotic genes such as IRF1 and CASP1, but also significantly reduces the sensitivity of tumor cells to cytokines secreted by immune effector cells (Chen Z. et al., 2023; Wang et al., 2020).

Furthermore, within the highly regulated cell death pathway of necroptosis, phosphatases form a complex, multi-layered regulatory network through the precise localization and dephosphorylation of core signaling molecules RIPK1 and RIPK3. This network is primarily characterized by inhibitory effects: for instance, the PP2A-B56α complex elevates the activation threshold of RIPK1 by dephosphorylating “inhibitory sites” on RIPK1 (Guffens et al., 2023; Saddoughi et al., 2013). Meanwhile, PPM1B directly targets RIPK3 by removing critical phosphorylations from its kinase domain, thereby inhibiting RIPK3 autophosphorylation and its recruitment of downstream MLKL (Chen et al., 2015). Additionally, the PP1γ-PPP1R3G holoenzyme plays a unique bidirectional regulatory role: it releases the “brakes” on RIPK1 by dephosphorylating its inhibitory sites, thereby promoting necroptosis during the initial signaling phase; however, during the later stages of RIPK1 hyperactivation, the same dephosphorylation process exerts negative feedback to limit its activity (Du et al., 2021).

### 4.3 Initiation of compensatory survival programs

When fundamental defense mechanisms are partially compromised, phosphatases swiftly activate deeper compensatory protective programs to ensure cell survival through autophagy, protein stabilization, and metabolic adaptation.

Under stressful conditions such as hypoxia, PP2A relieves mTORC1-mediated inhibition of the ULK1 complex by dephosphorylating its key components, thereby initiating protective autophagy (Migliore et al., 2024; Hajdu et al., 2022). This process not only degrades invading granzyme B but also eliminates mitochondria damaged by immune attacks, thereby suppressing the mtDNA leakage-activated cGAS-STING pathway and avoiding further immune recognition and activation (Zhang et al., 2021). Phosphatase PP1 and PP2A significantly enhance the stability of X-linked inhibitor of apoptosis protein (XIAP) by maintaining its specific dephosphorylated state, enabling sustained and effective inhibition of CASPASE-3, -7, and -9 activities (Gardai et al., 2004). This mechanism synergizes with the serine protease inhibitor SERPINB9, which effectively neutralizes the protease activity of granzyme B, collectively forming a dual inhibitory network targeting the core apoptotic execution phase (Teo et al., 2024). Low-molecular-weight phosphatases promote the remodeling of cellular membrane phospholipids by dephosphorylating and activating long-chain acyl-CoA synthetase 4, enhancing cellular resistance to oxidative stress (Alho et al., 2013; Lori et al., 2018). Meanwhile, members of this phosphatase family maintain the dephosphorylated state of the xCT subunit of the cystine/glutamate antiporter, ensuring efficient cystine uptake and collectively sustaining intracellular reduced glutathione levels, thereby effectively counteracting IFN-γ-induced ferroptosis (Huang et al., 2022).

### 4.4 Expansion of non-canonical protective networks

In addition to classical pathways, phosphatases also extend their cellular protective networks through non-canonical mechanisms such as transcriptional reprogramming and cell cycle arrest. Dephosphorylation of SMAD2/3 mediated by phosphatase PP2A promotes its nuclear translocation, thereby upregulating the expression of the transcription factor SOX4 (Prasad et al., 2024; Ballout et al., 2024; Petritsch et al., 2000). SOX4 significantly suppresses CTL-induced tumor cell apoptosis by regulating the expression of a series of anti-apoptotic genes, providing transcriptional support for tumor survival (Batut et al., 2008). Phosphatase PP1 maintains the dephosphorylated state of retinoblastoma protein (RB) family members, arresting the cell cycle in the metabolically less active G0/G1 phase (Verdugo-Sivianes and Carnero, 2021). This “dormant” state not only reduces the nutritional demands of tumor cells but also significantly enhances their survival capacity in harsh microenvironments characterized by hypoxia, nutrient deprivation, and immune pressure.

### 4.5 Summary

#### 4.5.1 Core mechanism

Phosphatases achieve ultimate cellular protection by constructing a multi-layered defense system that spans from physical barriers and death signal interception to the activation of proactive survival programs. This system exhibits a high degree of redundancy and adaptability, ensuring that even if some defensive lines are breached, cell survival can still be maintained through compensatory mechanisms.

#### 4.5.2 Existing controversies and challenges

The core challenge lies in distinguishing the different roles of phosphatases in tumor cell protection versus maintaining normal tissue homeostasis to achieve therapeutic specificity. Moreover, how various protective mechanisms (such as autophagy and apoptosis inhibition) are prioritized and dynamically switched through the phosphatase network remains an unresolved mystery. Additionally, the epigenetic regulatory role of phosphatases in the process by which tumor cells memorize and adapt to repeated immune attacks (i.e., “clonal evolution”) also requires urgent exploration.

#### 4.5.3 Therapeutic opportunities

For the “cellular protection” network, the most promising strategy is “multi-pathway synergistic blockade.” For instance, combining PP2A inhibitors (to disrupt physical barriers and autophagy) with SHP-1 inhibitors (to restore death receptor sensitivity) may generate a potent synergistic effect, completely dismantling the tumor’s last line of defense. Meanwhile, the combination of targeting phosphatases with ferroptosis inducers offers a new direction for overcoming apoptosis resistance.

## 5 Discussion

**FIGURE 1:**
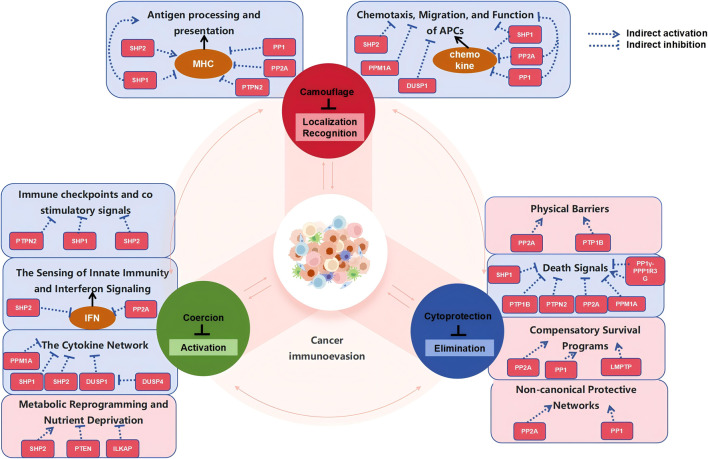
Phosphatase is a molecular switch in the “three Cs” framework of immune escape. The latest research has proposed a “three Cs” framework for immune evasion, wherein tumor cells achieve immune evasion through camouflage, coercion, and cytoprotection. Tumor cells evade immune recognition by downregulating antigen presentation and interfering with the chemotaxis and migration of immune cells. Besides affecting signal transduction in APCs, phosphatases primarily regulate key molecules involved in antigen recognition and chemokines indirectly, thereby facilitating immune “camouflage.” When the camouflage is partially uncovered, tumor cells shift to an active offensive strategy. They disrupt the stability of immune checkpoints, suppress innate immune and interferon signaling, reprogram cytokine networks, and orchestrate metabolic reprogramming and nutrient deprivation, directly suppressing the function of immune cells. When the immune system successfully recognizes and attacks tumor cells, phosphatases construct a final line of defense through multiple mechanisms, including mediating the reconstruction of physical barriers, activating compensatory survival signals, triggering protective network activation, and blocking death signals, thereby ensuring the survival and continued evasion of tumor cells. Throughout this process, multiple phosphatases are involved in multi-stage and multi-target regulation. Notably, the same phosphatase may exert opposing functions at different stages, underscoring the high degree of complexity of the phosphatase network in immune evasion.

Phosphatases construct a highly coordinated multi-Layered defense system within the “Camouflage, Coercion, and Cellular Protection” (three Cs) framework of tumor Immune evasion through their spatiotemporally specific dephosphorylation (Figure 1). This network initiates with “camouflage”: phosphatases achieve initial immune evasion by suppressing antigen presentation (e.g., PP2A-mediated autophagic degradation of MHC-I), blocking chemotactic signals (e.g., SHP-1-induced dephosphorylation of STAT1 inhibiting CXCL10 production), and enhancing “do not eat me” signals (e.g., SHP-2 regulation in the CD47-SIRPα axis). Transitioning to the “coercion” phase, phosphatases actively suppress immune cell function by stabilizing immunosuppressive ligands (e.g., PTPN2 regulation of PD-L1 degradation), disrupting danger signal transduction (e.g., PP2A inhibition of the cGAS-STING pathway), and remodeling the metabolic environment (e.g., CD73-mediated adenosine production). When confronted with direct immune attacks, phosphatases exert their ultimate defensive role in the “cellular protection” dimension: constructing a robust final line of defense by altering cellular mechanical properties (PP2A-mediated cytoskeletal reorganization), blocking death receptor signaling (SHP-1 inhibition of the FAS pathway), and initiating compensatory survival programs (e.g., autophagy activated by the PP2A-ULK1 axis).

However, in-depth research in this field faces three core challenges:

First, the spatiotemporal specificity and network complexity of phosphatase regulation remain far from fully understood. Phosphatase activity is finely regulated by subcellular localization, regulatory subunit combinations, post-translational modifications (e.g., redox sensitivity and acetylation), and microenvironmental signals (e.g., hypoxia/metabolic stress), yet current research methods struggle to capture such dynamic changes. More importantly, studies have predominantly focused on the unidirectional regulation of phosphatases, overlooking the “phosphorylation-dephosphorylation” dynamic equilibrium network they form with complementary kinases (e.g., PP2A-AKT, PTP1B-JAK). The global effects of this bidirectional dysregulation and its dynamic evolution during immune editing remain uncharted territories.

Second, tumor heterogeneity and the dual roles of phosphatase functions pose fundamental obstacles to therapeutic targeting. Significant differences exist in phosphatase networks across tumor types (e.g., highly immunogenic melanoma versus poorly immunogenic pancreatic cancer), yet systematic subtype studies are currently lacking. More critically, many phosphatases (e.g., SHP2, PP2A) play opposing roles in tumor cells and immune cells, making systemic targeting potentially suppress antitumor immunity simultaneously and leading to therapeutic efficacy offset or unintended toxicity.

Finally, the development of targeting strategies faces dual biochemical and pharmacological bottlenecks. The high conservation of catalytic domains within the phosphatase family makes designing highly selective small-molecule inhibitors exceedingly challenging, prone to off-target effects (e.g., the impact of SHP2 inhibitors on cardiac development) (Stanford and Bottini, 2017). Meanwhile, traditional drug development approaches have repeatedly failed due to the positively charged nature of protein phosphatase active sites and the lack of allosteric pockets (Table 1).

**TABLE 1:** Key gaps and future strategies in phosphatase research.

Research gaps	Specific challenges	Potential strategies and future directions
Insufficient awareness of network dynamism	The spatiotemporal regulatory mechanism of phosphatase kinase network is unclear	Develop new biosensors that combine live cell imaging and single-cell sequencing to track real-time phosphatase activity dynamics; constructing a mathematical model of regulatory network using systems biology methods
Dual functions in tumors and immune cells	Systemic inhibition may lead to therapeutic effect cancellation or immune toxicity	Developing precursor drugs responsive to the tumor microenvironment; explore strategies that target specific regulatory subunits (such as the B55 α subunit of PP2A) rather than catalytic cores; using nanocarriers to achieve local delivery of tumors
The difficulty of target conservation and selectivity	The catalytic domain is highly conserved, and the selectivity of inhibitors is poor	Shift towards the development of allosteric inhibitors (such as SHP2 allosteric inhibitor SHP099); selective degradation of specific phosphatases using PROTAC technology; exploring intervention strategies for phosphatase substrate interaction interface
Limitations of clinical translation models	The existing models cannot reflect the complexity of the human tumor immune microenvironment	Establish humanized mouse models and 3D organoid co culture systems to better simulate tumor immune interactions; conduct biomarker research based on multi omics for patient stratification

Targeting phosphatase activity within the “three Cs” framework holds core therapeutic significance in potentially achieving “single-target, multi-effect” synergistic therapy. However, this necessitates transcending traditional “complete inhibition” paradigms and shifting toward more refined “precision modulation.” Future breakthrough strategies may include:

Temporal Intervention Strategies: Selecting distinct targets based on therapeutic stages—early-stage utilization of PTPN2 inhibitors to enhance antigen presentation and reduce PD-L1 expression (dismantling camouflage), mid-stage combination of SHP2 and CD73 inhibitors to reverse metabolic suppression (alleviating coercion), and late-stage application of PP2A or SHP-1 inhibitors to dismantle cellular protection mechanisms.

Synergistic Combination Paradigms: Rationally combining phosphatase-targeted agents with existing immunotherapies. For instance, PTPN2 inhibitors may synergize with anti-PD-1 therapy by simultaneously enhancing immune recognition and blocking compensatory checkpoints; SHP2 inhibitors could be combined with adoptive T-cell therapy to improve T-cell functionality within immunosuppressive microenvironments.

Novel Modality Exploration: Developing PROTAC degraders, allosteric modulators, or therapies targeting specific regulatory subunits of “undruggable” phosphatases to address selectivity challenges.

## 6 Conclusion

In summary, phosphatases act as “molecular switches” for tumor immune evasion within the “three Cs” framework by precisely regulating protein dephosphorylation: achieving immune stealth during camouflage, executing active suppression in coercion, and establishing an ultimate survival defense in cytoprotection. Analyzing this multi-dimensional regulatory network not only unveils the systemic nature of tumor immune evasion but also underscores the pivotal therapeutic value of phosphatases as targets. Future research must bridge the knowledge gap from single-pathway inhibition to dynamic network reprogramming. By integrating systems biology with precision intervention strategies, we can overcome current targeted therapy bottlenecks and ultimately realize a “one target, multiple effects” immunotherapy paradigm based on the phosphatase regulatory network.
